# NICOTINAMIDE RIBOSIDE SUPPLEMENTATION PROTECTS AGAINST VIRAL-INDUCED NEURODEGENERATION IN AGED MICE

**DOI:** 10.1093/geroni/igae098.2632

**Published:** 2024-12-31

**Authors:** Daniel Enterria-Morales, Anais Claire Gonzalez, Bianca Jallorina, Adrian Martinez, Andrew Doan, Aadit Karve, Mikaela Joya, Matthew Shtrahman

**Affiliations:** University of California San Diego, La Jolla, California, United States; University of California San Diego, La Jolla, California, United States; University of California San Diego, San Diego, California, United States; University of California San Diego, San Diego, California, United States; University of California San Diego, San Diego, California, United States; University of California San Diego, San Diego, California, United States; University of California San Diego, San Diego, California, United States; University of California San Diego, La Jolla, California, United States

## Abstract

Several lines of research support the hypothesis that boosting cellular nicotinamide adenine dinucleotide (NAD+) may induce metabolic adaptation that helps neurons maintain healthy function as they age and slow neurodegeneration, making this approach a promising therapy for neuroregeneration. To prove this strategy, we tested the ability of Nicotinamide Riboside (NR), a pyridine-nucleoside precursor to NAD+, to prevent neuronal cell loss in a model of viral induced neurodegeneration using recombinant adeno-associated virus (rAAV). C57BL/6J wild-type mice (18 months old) were obtained from the National Institute of Aging (NIA). NR was administered into the animals’ drinking water (12mM) for three consecutive weeks before the injection of a recombinant rAAV expressing the LacZ gene under the control of the CMV promoter (rAAV-CMV-LacZ) at a dose of 1x1013 gc/mL into the unilateral hippocampus. For the control groups, the mice were injected either with a saline solution or with the rAAV-CMV-LacZ, but no NR supplementation was provided. For the experimental group, NR treatment was maintained for 12 weeks until the date of sacrifice. We found that NR supplementation partially rescued the rAAV-induced neurodegeneration in the CA3 of the hippocampus. NR supplementation reduced the activation of microglia and astrocytes in the CA3, partially rescuing the inflammatory response to the rAAV infection. Our findings support the idea of boosting NAD+ levels as a neuroprotective approach to metabolically help neurons of the hippocampus to respond to chronic damage in the form of aging. Further mechanistic studies are required to better understand the cellular pathways that underlie these findings.

